# Formation of hollow silver nanoparticles under irradiation with ultrashort laser pulses

**DOI:** 10.1515/nanoph-2023-0881

**Published:** 2024-02-16

**Authors:** Francisco Sánchez-Pérez, Olivia Borrell-Grueiro, Alfredo Casasnovas-Melián, Diego J. Ramos-Ramos, Andrés Guerrero-Martínez, Luis Bañares, Alejandro Prada, Felipe J. Valencia, Jorge Kohanoff, Miguel L. Crespillo, José Olivares, Antonio Rivera, Ovidio Peña-Rodríguez

**Affiliations:** Instituto de Fusión Nuclear “Guillermo Velarde”, Universidad Politécnica de Madrid, José Gutiérrez Abascal 2, E-28006, Madrid, Spain; Departamento de Química Física, Universidad Complutense de Madrid, Avenida Complutense s/n, 28040, Madrid, Spain; INSTEC, Universidad de la Habana, Avenida Salvador Allende 1110, 6163, 10400, Habana, Cuba; Instituto Madrileño de Estudios Avanzados en Nanociencia (IMDEA Nanoscience), Cantoblanco, 28049, Madrid, Spain; Departamento de Computación e Industrias, Facultad de Ciencias de la Ingeniería, Universidad Católica del Maule, Talca, Chile; Centro de Micro-Análisis de Materiales, Universidad Autónoma de Madrid, Madrid, E-28049, Spain; Instituto de Óptica “Daza de Valdés” (CSIC), Serrano 121, Madrid, E-28006, Spain; Departamento de Ingeniería Energética, ETSII Industriales, Universidad Politécnica de Madrid, José Gutiérrez Abascal 2, E-28006, Madrid, Spain; Centro de Innovaciön en Ingeniería Aplicada (CIIA), Facultad de Ciencias de la Ingeniería, Universidad Catölica del Maule, Talca, Chile

**Keywords:** hollow silver nanoparticles, amorphous silica, irradiation of plasmonic nanostructures, ultrashort laser pulses, molecular dynamics

## Abstract

We have studied the formation of cavities in spherical silver nanoparticles embedded in silica, irradiated with fs laser pulses that produce an intense electronic excitation. Experimentally determined aspect ratio, i.e. the ratio between the cavity and nanoparticle size, for hollow structures formed under different irradiation conditions shows a very good agreement with values obtained by means of atomistic simulations. According to the predictions of the atomistic model, one can produce at will hollow silver nanoparticles with cavities of tailored dimensions, having an accurate control. Hence, laser irradiation can be used to control and design the optical response by tuning the localized surface plasmon resonances of the hollow nanoparticles.

## 1 Introduction

Metallic nanoparticles (NPs) have generated much interest in recent years, due to their remarkable optical properties. The main reason for this attention is the presence of localized surface plasmon resonances (LSPRs) [1], [2], [3], which are collective oscillations of conduction band electrons, and the flexibility that they offer to have an accurate control on the optical properties [4], [5], [6]. In turn, this opens the door to a multitude of applications such as catalysis [7], [8], [9], [10], [11], [12], [13], biological and chemical sensing [14], [15], [16], [17], [18], biological imaging [19], photonics and energy harvesting and production [20], [21], [22], [23], [24], [25], [26], dye-sensitized solar cells [27], storage [28], including hydrogen storage [29], [30], surface enhanced spectroscopies [31], [32] and even for medical therapies like cancer treatment [33], [34] or drug and gene delivery and therapeutics [35], [36], [37], [38].

Among the different nanoparticle types, hollow nanospheres (nanoshells) are very interesting, due to the possibility of controlling their LSPR by means of the geometrical parameters, namely, the aspect ratio (i.e. the ratio between the inner and outer radii) [39]. Consequently, there are numerous studies about their manufacturing by chemical processes [27], [40], [41], [42] and through physical processes such as laser ablation [43], [44]. More recently, it has been proposed that ultrashort laser pulses can be used to fabricate hollow NPs [45], [46], [47]. Molecular dynamics (MD) simulations have become a very useful tool to analyse the dynamics of NP at the atomic scale and further understand the process of cavity formation. Moreover, those studies have identified the conditions required for cavity formation in NPs: a fast, nearly adiabatic heating, followed by a swift quenching. The former condition is easy to meet experimentally, using ultrashort laser pulses, but the latter is harder to achieve, particularly for colloidal NPs [45].

In this work, the formation of cavities is demonstrated experimentally by irradiating silver NPs embedded in silica with femtosecond laser pulses that produce an intense electronic excitation. This irradiation, through heating, expands the NPs generating inner cavities, evidenced by the changes observed in the LSPR frequency. Simultaneously, MD simulations have been carried out to explain the mechanism of cavity formation. In particular, the effects produced by a single laser pulse on a silver NP depending on its size and laser fluence, as well as the temporal evolution of the NP’s temperature and aspect ratio, have been studied in detail.

## 2 Experiment

**Figure 1:**
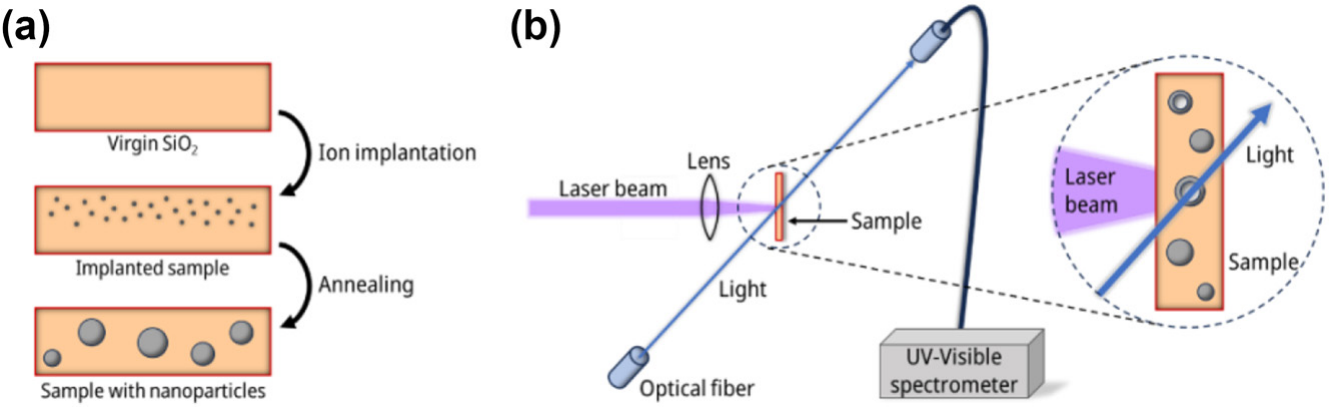
Sample fabrication and in situ measurements. (a) Schematics for the manufacturing process of silver NPs in silica: a silica glass substrate is implanted with 150 keV Ag^+^ ions in a shallow layer (<100 nm) near the surface, and then subsequent thermal annealing process induces the nucleation of silver nanospheres. (b) Scheme of the experimental setup for the formation of cavities in silver NPs. The sample is irradiated by 400 nm laser pulses and the irradiation fluence is regulated by shifting the sample position with respect to the optical lens. The *in situ* optical absorption spectra are collected from the sample simultaneously to the irradiation process.

High-purity silica glass plates (60 × 60 × 0.5 mm^3^) were implanted at room temperature with 150 keV Ag ions at a fluence of 1 × 10^17^ cm^−2^, using the 210 kV Ion implanter (Danfysik) at Campus Tecnológico e Nuclear, Instituto Superior Técnico (Lisbon, Portugal) [48]. Ag ions were implanted in a shallow layer (<100 nm) near the surface. Afterwards, the samples were cut into smaller pieces and annealed in air for an hour, at a temperature of 600 °C, to induce the nucleation of silver nanospheres (Figure 1(a)) [49], [50].

After the thermal treatment, the samples were irradiated with ultrashort laser pulses (100 fs) for 60 min, at a frequency of 1 kHz, and using different fluences: 24, 27 y 31 J/m^2^ (lower fluences were performed as well, but no apparent changes in the optical properties were observed). Fluence (*ϕ*) was calculated through the relationship *ϕ* = *E*/*A*, where *E* is the energy of each pulse (in this case, *E* was 1.5 mJ for the second harmonic), and *A* the beam area over the sample. The diameter of the unfocused beam (15 mm) was determined by projecting it on a graph paper. Then, we used a lens with a focal distance of 500 mm to focus the laser beam, and the fluence was controlled by changing the distance between the lens and the sample. The fluences reported in this work correspond to distances of 200, 220 and 240 mm (beam diameters of 9.0, 8.4 and 7.8 mm, respectively).

The irradiation experiments were conducted at CMAM (Centro de Micro-Análisis de Materiales) [51], using a Ti:Sapphire femtosecond laser (Spectra Physics Solstice ACE model, regenerative amplifier). The laser emits 100 fs pulses, with a repetition rate of 1 kHz, and a wavelength of 800 nm. The second harmonic of the laser (400 nm) was used to match the LSPR wavelength of spherical silver NPs. Simultaneously, the *in situ* optical absorption spectra of the samples were collected using a UV-visible spectrometer (QE6500, Ocean Optics Inc.), at different times during the irradiation, to analyse the changes in the optical response as a function of time and fluence (Figure 1(b)).

## 3 Molecular dynamics

To better understand the experimental results, the irradiation process was simulated using molecular dynamics (MD), with the LAMMPS code [52]. MD using classical force fields cannot address the relevant electronic processes involved, such as, plasmon formation, plasmon decay or electron–phonon coupling, but, due to the difference in timescales between the energy transfer from the plasmon to the atomic system and the atomic lattice evolution, it is possible to obtain meaningful results decoupling both effects. Namely, it is assumed that the energy is transferred from the laser beam to the electron system in a very short time (∼100 fs), and then the energy is transferred to the atomic lattice in a timescale of picoseconds, ignoring the details of energy transfer from the electrons to the atoms, as we have done with satisfactory results in recent works [45], [53]. This approach allows the use of a simple atomistic model based on classical force fields to study the formation of cavities in silver NPs.

A variety of force fields to represent the interaction between different types of atoms was used. The interaction between the atoms in silica (i.e. Si–Si, Si–O and O–O interactions) was described via a Tersoff potential [54] as its thermal conduction is in good agreement with previous experimental results [55]. On the other hand, an EAM potential was used for the Ag–Ag interaction [56]. Although this potential was made for systems at room temperature (300 K), subsequent works have studied its use for cases where silver nanoparticles reach the melting temperature, comparing it with experimental results and literature, reaching a good agreement [57], [58]. Moreover, there are works that use it for melting and coalescence studies with other metals, mixing silver with palladium [59] or copper [60]. Finally, a Lennard-Jones (LJ) potential to simulate the interaction of the metallic NP with the silica (Ag–Si and Ag–O interactions) [61].

The initial state was defined as a box of crystalline silica with a cristobalite structure of 30 × 30 × 30 nm^3^. Once the lattice was formed, the box was subjected to various thermal treatments (raising its temperature up to 7000 K and decreasing it in steps of 1000 K every 25 ps until 300 K) until a state of amorphization at room temperature is reached [62]. Next, around 34,000 atoms were extracted from a spherical region in the centre, preserving the stoichiometry of the system, and the hole was filled with Ag atoms until a spherical crystalline Ag NP of 5 nm radius with more than 30,000 atoms was formed. A small gap of 0.1 nm was left between the Ag atoms and the silica, so that, after a slight annealing (raising the system temperature up to 600 K in 25 ps, maintaining it for another 25 ps, decreasing it again to 300 K in 25 ps and maintaining it for 25 ps), as already carried out in previous works [63], the sample was completely relaxed. The dimensions of the system have been chosen in such a way that they lie between those of irradiated NPs, allowing the formation of cavities of appreciable size to be analysed, but optimizing computational resources.

Once the system was relaxed (at 300 K), a series of simulations, increasing linearly the temperature of the silver NP for 7 ps in order to roughly reproduce the electron–phonon coupling [45], up to a desired temperature were performed, covering a wide range of temperatures from 1000 to 5000 K. Immediately, after this temperature was reached, the system was allowed to relax naturally, in the microcanonical ensemble (NVE). The silica surrounding the NP removes heat from it, resulting in an effective cooling-down of the NP. The maximum temperature reached by the nanoparticle depends on the laser fluence and its size, i.e. the larger the laser fluence, the higher the temperature it reaches, whereas for larger particles, where their absorption cross section is dominated by scattering, the temperature reached will be lower. Finally, the results obtained with these simulations were compared with the optical results.

## 4 Results and discussion

**Figure 2:**
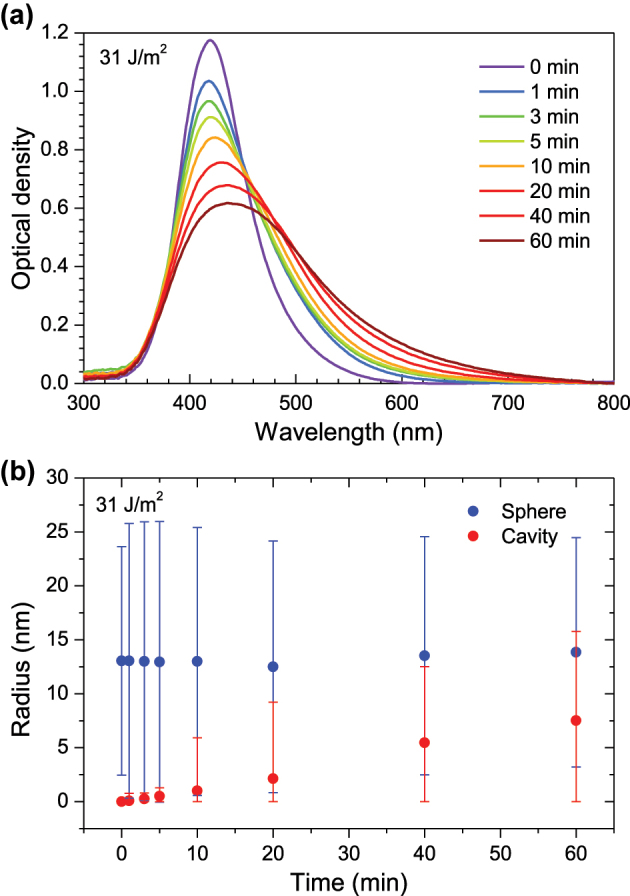
Optical absorption spectra and particle sizes. (a) Time evolution of the optical absorption spectra for silica samples containing silver NPs, irradiated with 400 nm fs laser pulses with a frequency of 1 kHz and a fluence of 31 J/m^2^. (b) Nanoparticle (blue) and cavity (red) radii, as a function of time, for a fluence of 31 J/m^2^. Error bars in panel (b) represent the standard deviation of the distribution used to represent the cavity and particle radii. Standard deviation of NP radius appears to reduce over time, which could be due to Ostwald ripening [66] (smaller nanoparticles are disintegrated by the laser pulses, being absorbed by larger nanoparticles).

**Figure 3:**
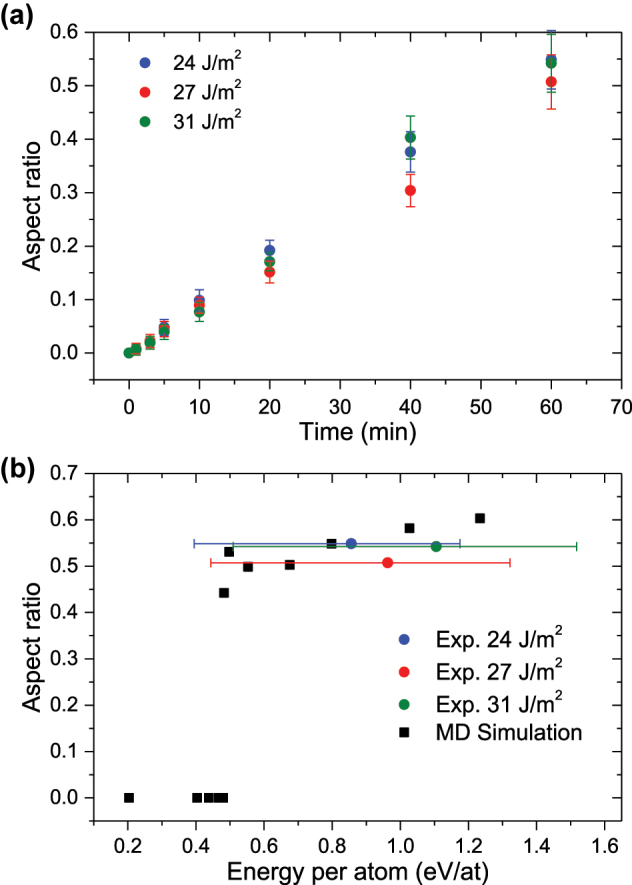
Determination of aspect ratio. (a) Average aspect ratio of the hollow silver NPs, as a function of fluence and time, obtained from a fit of the optical absorption spectra. (b) Aspect ratio as a function of the energy absorbed per Ag atom from the experimental results (blue, red and green; after irradiating the samples for 60 min) and those obtained by MD simulations (black), showing values between 0.45 and 0.60. For the simulations, a discontinuity is observed when the energy per atom reaches 0.5 eV. The horizontal error bars show the energy absorbed per atom of larger NP (left), which has a dominant scattering term, and of smaller NP (right), whose absorption term is larger than the scattering term.

The evolution of the optical absorption spectra for the sample irradiated with a fluence of 31 J/m^2^ is depicted in Figure 2(a) (the spectral changes for the other fluences are similar). It is clear from these data that the LSPR of some of the silver NPs, initially around 410 nm, is considerably redshifted upon irradiation (up to 550 nm) and, in addition, the plasmon peak widens significatively. However, the overall plasmon intensity does not decrease, which suggests that there is not significant disappearance of NPs, just structural modification. For a more quantitative assessment, the experimental spectra were fitted with the MieLab tool [64] and following the procedure that we have described previously [65]. For each laser fluence, all the relevant geometrical parameters were extracted from this fit, namely: the cavity radius and the average and standard deviation for NP size (Figure 2(b)). Finally, the aspect ratio can be calculated as a function of irradiation time (Figure 3(a)).

Now, the initial properties of the irradiated samples must be considered to understand the experimental results. As described elsewhere [63], NPs fabricated by ion implantation present a large size dispersion. Particles with different sizes have dissimilar absorption cross sections and stability and, hence, their interaction with the laser beam is not equal. For instance, optical absorption is dominant for smaller NPs, whereas scattering is more important for larger ones. Thus, a complex kinetics with at least three different behaviours is expected. First, very small particles (radii below 2–3 nm) are probably disintegrated by the laser pulse and its atoms can later regroup to form a new NP or be absorbed by larger ones (Ostwald ripening [66]). Second, the larger particles (*R* > 20 nm) are barely affected by the laser pulses (in the irradiation regime used in this work). Finally, particles with intermediate sizes are expanded by the laser pulse, forming cavities. The combined effect of these three types of behaviour can be observed in Figure 2(a), where some NPs form cavities, red shifting their plasmon wavelength, whereas others remain as solid nanospheres, and, consequently, their plasmon band is not affected by the irradiation. After 1 h of irradiation, the absorption spectra reached a relatively stable state, suggesting that some of the particles have been modified significantly (i.e. a cavity was produced) and no longer interact with the laser beam, whereas other particles are too stable and, hence, are not affected by the laser pulses.

**Figure 4:**
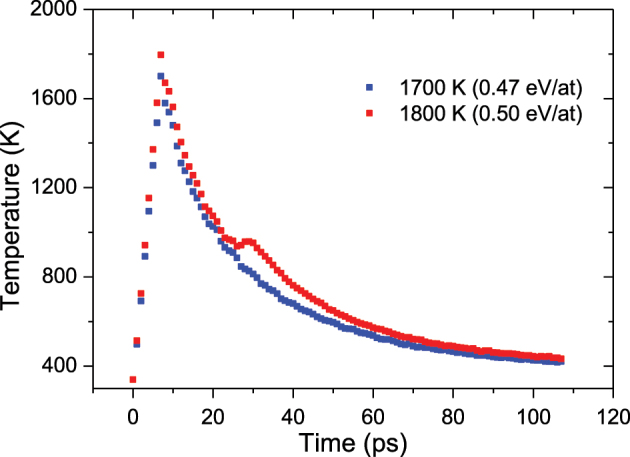
Time evolution of silver NP temperature when the temperature is increased to 1700 K, i.e. 0.47 eV/at, (blue) and when the temperature is increased to 1800 K, i.e. 0.50 eV/at (red). Both present similar decay time constants, but in the case of 1800 K, a plateau appears that corresponds to the moment of cavity formation (∼20 ps).

To understand the observations, a comparison between the optical results and those obtained with the atomistic model has been conducted (Figure 3(b)). The comparison provides an in-depth detailed explanation on the underlying mechanisms for the formation of hollow NPs. Due to the NPs’ size dispersion, the energy absorbed per atom differs considerably for the larger and smaller NPs and has been represented as error bars in Figure 3(a) (the symbols represent the energy absorbed by the NPs with an average size). Scattering dominates over absorption when the NPs are larger, so the energy absorbed per atom will be lower and the formation of cavities may be due to a cumulative process of energy, produced by several pulses. On the other hand, for smaller NPs, the absorption is dominant, so that they will absorb more energy per atom, and a single pulse could be enough to form a cavity. For instance, MD data give the temporal evolution of the temperature of the silver NP. As can be observed (Figure 4), the temperature of the NP drops following an exponential decay, with time constants ranging from 25 ps (in the simulations where the NP acquires a higher temperature) to 35 ps. An interesting observation is the appearance of a small plateau in the temperature evolution, in coincidence with the formation of the cavity. Following this plateau, a small peak also appears. These effects seem to be related to the cavity formation, where atoms with high kinetic energy move radially, leaving an empty space inside. During this process, there is hardly any energy transfer from the silver atoms to the surrounding silica, which flattens the temperature curve. This plateau appears for lower fluences, when the initial temperature after heating is relatively low (between 1750 and 2000 K), but when the maximum temperature reached by the NP is higher, the cavity is formed earlier and at a higher temperature. Hence, the energy transfer from the NP to the silica matrix is larger, making that plateau almost negligible.

To compare the optical data with MD simulations, the total energy deposited per atom was calculated for both cases. For MD simulations, the energy deposited during the 7 ps of temperature increase was determined and divided by the total number of Ag atoms in the NP. In the experimental case, the absorption cross section was obtained with MieLab [64], using the size of the NPs obtained from the fit. Then, the energy absorbed by the NP (*E*_*abs*_) can be obtained using the absorption cross section (*σ*_*abs*_), the laser fluence (*F*), the nanoparticle’s volume (*V*), and the atomic density of silver (*ρ*): $E_{\mathrm{abs}}=\left(F\cdot \sigma_{\mathrm{abs}}\right)/\left(V\cdot \rho \right)$ .

**Figure 5:**
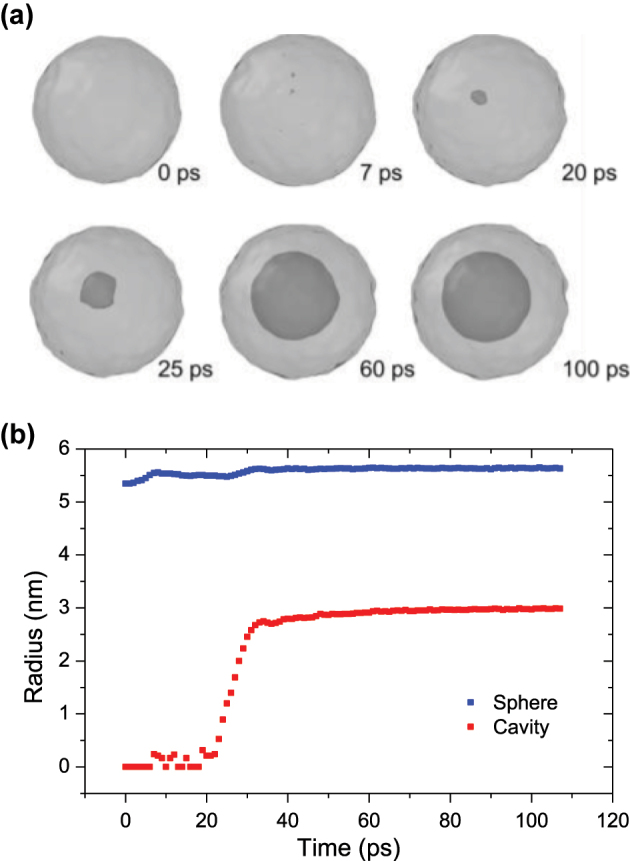
Formation of hollow nanoparticles. (a) Snapshots of a silver NP and the corresponding cavity at different times, showing relevant stages: the initial state (0 ps); moment of expansion that matches the maximum temperature reached by the NP (1800 K; 7 ps); the beginning of cavity formation (20 ps and 25 ps); and, when the stationary stage is reached, the NP shape and cavity size barely change (60 ps and 100 ps). The snapshots were generated with Ovito visualization software [67]. (b) Temporal evolution of the radius of the same silver NP and the corresponding cavity. It is observed how in the initial moments the NP expands slightly (from 5.34 nm to 5.55 nm). After approximately 20 ps from the onset, a rapid formation of the cavity begins. Finally, the cavity reaches a size that barely varies over time (∼3 nm) with a sphere radius of 5.63 nm.

MD simulations show the temporal evolution of the formation of the inner cavity, as can be observed in Figure 5. Thus, after the initial laser pulse, the NP expands slightly, whereas its temperature increases to its maximum at 7 ps, producing a large mobilization of the NP atoms (firsts two snapshots in Figure 5(a)). However, the appearance of the cavity does not occur until several picoseconds (∼20 ps) have passed (third snapshot in Figure 5(a)), during which the NP expands rapidly and then transfers its energy to the silica atoms, stabilizing the size of the cavity (latest snapshot in Figure 5(a)). From this time onwards, the cavity size increases very slightly until it reaches a stable state, around 100 ps (Figure 5(b)). Furthermore, MD simulations show a clear threshold for cavity formation, around 0.5 eV/atom (Figure 3(b)). Moreover, they are in very good agreement with the experimental data (Figure 3(b)), showing that, regardless of the amount of energy absorbed by the NPs, hollow NPs always have similar aspect ratios, between 0.45 and 0.60.

In the following, the mechanism of cavity formation will be discussed. It is noticeable that for NPs embedded in silica, the cavity formation during the expansion is not reversed, as can occur when the NP is in vacuum [45]. In the case of vacuum, NPs can expand without resistance, and afterwards they can contract again since there is no medium that prevents these displacements. However, when the NP is embedded in a solid matrix such as silica, this material limits considerably its expansion (Figure 5), but, despite this limitation, the silica seems to interact with the outermost layers of silver atoms and prevents the cavity collapse returning to the initial solid sphere. These conclusions have arisen from MD simulations. For other particle sizes, especially large ones, where the scattering term predominates over absorption, the cavity formation mechanism might not be produced by a single pulse, but from the accumulation of energy, deposited by several successive pulses. This effect could be analysed in the future, by irradiating samples containing NPs with a narrow size dispersion and performing MD simulations of large ones.

## 5 Conclusions

It has been experimentally observed that the irradiation of silver NPs under intense electronic excitation with fs laser pulses produces cavities inside them, producing a redshift of the LSPR band. These results were compared with MD simulations that yield comparable results, indicating that the proposed atomistic model is a powerful tool to predict the evolution of cavity formation in silver NPs embedded in silica. This model provides additional insight into the temporal evolution of the formation of the cavities, which start with an expansion of the NP induced by the severe atom motion preferentially outwards in radial direction. This leads to the formation of a cavity and a final stabilization of the system due to the fast temperature quenching. In addition, it provides information on how temperature evolves over time, decaying exponentially with time constants of the order of 30 ps. It has been observed that silica plays a key role, constraining further expansion of the NP but later helping to stabilize the cavity. This study shows an alternative methodology to produce hollow NPs with accurate size-control for use in a wide range of industrial and technological applications. Moreover, the results shown in this work might be extended to other metals, since it should be relatively simple to use the same route to create cavities in materials like gold, palladium or platinum, or using different ceramic host matrices.
